# Impacts of different dietary soybean meal levels on jejunal immunity of nursery pigs at different days post-weaning

**DOI:** 10.1186/s40104-025-01271-0

**Published:** 2025-10-24

**Authors:** Hyunjun Choi, Zixiao Deng, Sung Woo Kim

**Affiliations:** Department of Animal Science, North Carolina State University, Raleigh, NC 27695 USA

**Keywords:** Days post-weaning, Jejunal immunity, Nursery pigs, Soybean meal

## Abstract

**Background:**

The objective of this study was to investigate the impacts of different dietary soybean meal (SBM) levels on jejunal immunity in nursery pigs at different days post-weaning.

**Methods:**

Forty-eight pigs (6.2 ± 0.3 kg), weaned at 21 days of age, were assigned to 2 dietary treatments (*n* = 12) in a randomized complete block design and fed for 20 or 42 d in 3 phases (10, 10, and 22 d, respectively). The dietary treatments consisted of low and high SBM diets. On d 20 and 42, jejunal mucosa and tissue samples were collected. Treatments were arranged in 2 × 2 factors with dietary SBM levels (low and high SBM diets) and days post-weaning (20 d and 42 d post-weaning).

**Results:**

Pigs fed high SBM diets had greater (*P* < 0.05) relative abundance (RA) of jejunal *Prevotella*, tended to have greater (*P* = 0.091) jejunal IgA, had greater (*P* < 0.05) crypt depth, and tended to have lower (*P* = 0.064) villus height to crypt depth ratio (VH:CD) than pigs fed low SBM diets. Pigs at 20 d post-weaning had greater (*P* < 0.05) RA of jejunal *Lactobacillus* and had greater (*P* < 0.05) jejunal IL-8 and protein carbonyl than pigs at 42 d post-weaning. Pigs at 20 d post-weaning tended to have greater (*P* = 0.090) jejunal IgG, tended to have lower (*P* = 0.059) jejunal IgA, and had greater (*P* < 0.05) proportion (%) of Ki-67^+^ cells in the jejunal crypt than pigs at 42 d post-weaning.

**Conclusion:**

Pigs fed high SBM diets showed greater RA of *Staphylococcus*, a greater immune response, and a decreased VH:CD in the jejunum than pigs fed low SBM diets. Pigs at 20 d post-weaning were more susceptible to jejunal inflammation and intestinal damage than pigs at 42 d post-weaning, but the negative impacts of high SBM diets on jejunal inflammation and intestinal damage were consistent compared to low SBM diets at 20 d and 42 d post-weaning.

**Supplementary Information:**

The online version contains supplementary material available at 10.1186/s40104-025-01271-0.

## Background

Soybean meal (SBM) is a cost-effective and widely used protein supplement in pig diets due to its high amino acid content, high digestibility, and well-balanced amino acid profile [1–3]. However, in nursery pigs, the increased use of SBM raises concerns due to its anti-nutritional compounds, such as allergenic proteins (e.g., glycinin and β-conglycinin), soluble non-starch polysaccharides (NSP), and raffinose-family oligosaccharides [4–6]. These compounds negatively affect intestinal health and growth in nursery pigs [5, 7]. In this context, the jejunum is a critical site for intestinal health and growth in pigs, as it is the primary location for nutrient absorption and is where the composition of jejunal mucosa-associated microbiota rapidly changes in response to dietary SBM [8]. The jejunal mucosa-associated microbiota also directly interact with the mucus layer and intestinal epithelial cells through pattern recognition receptors and immune cells [9–11], making the jejunum a key site of host-microbiota interaction. Therefore, jejunal mucosa-associated microbiota are essential components of intestinal health, which is closely linked to the growth performance of nursery pigs in response to dietary SBM [6, 12]. 

Weaning represents the most vulnerable period in a pig’s life, during which environmental, immunological, and nutritional stressors disrupt the intestinal microbiota, impair intestinal health, and reduce growth [13, 14]. Among the stressors associated with weaning, nutritional stress is an influential component, as diet composition greatly affects the mucosa-associated microbiota and intestinal health of nursery pigs [15, 16]. In the early nursery phase (0 to 20 d post-weaning), pigs have immature intestinal mucosa and immune systems, making them highly sensitive to changes in dietary composition [17]. In contrast, in the later nursery phase (21 to 42 d post-weaning), pigs gradually adapt to weaning stress, leading to stabilization of the intestinal microbiota and maturation of both intestinal barrier function and immune responses. To address these concerns, SBM is often partially replaced with animal protein supplements or processed SBM during the early post-weaning period, whereas the inclusion rate of SBM increases during later stages [12, 18]. The physiological adaptations that occur as pigs age help mitigate the negative impacts of SBM and enhance the pig’s ability to tolerate its inclusion in the diets [19], potentially suggesting that pigs may be more susceptible to dietary SBM in the early nursery phase compared to the later nursery phase [12, 20]. Based on the previous findings, this study was hypothesized that nursery pigs would exhibit greater jejunal immune responses when fed high-SBM diets compared to low-SBM diets, particularly at earlier days post-weaning due to intestinal immaturity and increased susceptibility to anti-nutritional compounds in SBM compared to later days post-weaning. To test this hypothesis, the objective of this study was to investigate the impact of different dietary SBM levels on the jejunal mucosal microbiota and jejunal immunity in nursery pigs at 20 d and 42 d post-weaning.

## Materials and methods

The protocol of this experiment was reviewed and approved by North Carolina State University Animal Care and Use Committee (Raleigh, NC, USA).

### Animals, experimental design, and experimental diets

A total of 48 pigs (24 barrows and 24 gilts) weaned at 21 days of age with an initial body weight (BW) of 6.2 ± 0.3 kg were assigned to 2 dietary treatments in a randomized complete block design with initial BW and sex as blocks. Each treatment had 12 replicates (6 pens with barrows and 6 pens with gilts). Pigs were individually housed in pens (1.50 m × 0.74 m) and had free access to feed and water throughout the experimental period. High SBM diets contained 14% more SBM than low SBM diets. On d 20 and 42, pigs were euthanized to collect jejunal mucosa and tissues for jejunal immunity. Treatments were arranged in 2 × 2 factors with dietary SBM levels (low and high SBM diets) and days post-weaning (20 d and 42 d post-weaning). All the feedstuff used in this study originated from the same batch from North Carolina State University Feed Mill Education Unit (Raleigh, NC, USA). Experimental diets were formulated to meet or exceed the nutrient requirements suggested by NRC [21], except for metabolizable energy (ME), standardized ileal digestible (SID) Lys, SID Met, SID Thr, and SID Trp, total Ca, and apparent total tract digestible (ATTD) P in 3 phases (Table 1). The nutrient composition of the diets was lower than the nutrient requirements suggested by NRC [21], with reductions in ME (63 kcal/kg), SID Lys (140 mg/kg), SID Met (42 mg/kg), and SID Thr (84 mg/kg), total Ca (1,700 mg/kg), and ATTD P (1,360 mg/kg), respectively, accounting for phytase effects [22]. The experimental diets were supplemented with 1,000 or 3,000 FTU/kg of phytase, with equal numbers of replicates represented per level of SBM, to ensure balance within the dietary treatments. The experimental diets were provided as mash form. No antibiotics were included in diet. Pigs fed for 42 d in 3 phases: phase 1 (d 0 to 10), phase 2 (d 10 to 20), and phase 3 (d 20 to 42).

**Table 1:** Composition of low and high soybean meal (SBM) diets (as-fed basis)

Item	Phase 1		Phase 2		Phase 3	
	Low SBM	High SBM	Low SBM	High SBM	Low SBM	High SBM
Feedstuff, %						
Corn (yellow dent)	44.24	39.21	56.33	51.24	70.13	62.59
SBM	16.00	30.00	17.00	31.00	20.50	34.50
Whey permeate	20.00	20.00	12.00	12.00	-	-
Processed SBM^1^	6.00	-	6.00	-	6.00	-
Fish meal	4.00	3.00	2.00	2.00	-	-
Poultry meal	3.00	2.00	2.00	-	-	-
Blood plasma	4.00	3.00	1.50	-	-	-
Poultry fat	-	-	-	0.55	-	-
L-Lys HCl	0.37	0.31	0.45	0.42	0.46	0.24
DL-Met	0.17	0.16	0.17	0.17	0.14	0.08
L-Thr	0.13	0.10	0.14	0.14	0.13	0.04
L-Val	-	-	0.04	0.04	-	-
Limestone	0.70	0.82	0.98	1.06	1.11	1.05
Dicalcium phosphate	-	-	-	-	0.15	0.12
Mineral premix^2^	0.14	0.14	0.14	0.14	0.14	0.14
Vitamin premix^3^	0.03	0.03	0.03	0.03	0.03	0.03
Sodium chloride	0.22	0.22	0.22	0.22	0.22	0.22
Supplement (phytase + corn)	1.00	1.00	0.60	0.60	0.60	0.60
Titanium dioxide	-	-	-	-	0.40	0.40
Calculated composition						
Dry matter, %	91.63	91.48	90.24	90.12	88.79	88.75
ME^4^, kcal/kg	3,377	3,339	3,339	3,326	3,317	3,296
Crude protein, %	23.28	24.05	20.82	21.22	19.60	21.99
SID^5^ Lys, %	1.487	1.487	1.336	1.336	1.217	1.217
SID Met + Cys, %	0.816	0.816	0.735	0.735	0.676	0.676
SID Thr, %	0.872	0.872	0.781	0.781	0.722	0.722
SID Trp, %	0.251	0.266	0.215	0.223	0.202	0.238
SID Val, %	0.95	0.956	0.859	0.859	0.78	0.866
Total Ca, %	0.68	0.68	0.63	0.63	0.53	0.53
ATTD^6^ P, %	0.378	0.341	0.261	0.231	0.154	0.154
Analyzed composition, %						
Dry matter	89.37	89.29	88.08	87.88	87.30	87.16
Crude protein	23.25	23.40	18.21	19.73	18.22	20.75
Ether extract	2.82	2.64	3.15	3.11	3.00	2.89
Neutral detergent fiber	6.24	5.73	4.45	4.58	6.07	7.53
Acid detergent fiber	2.14	2.33	2.08	2.37	3.30	3.76
Calcium	0.94	0.98	0.74	0.72	0.60	0.61
Phosphorus	0.60	0.59	0.44	0.44	0.37	0.40

^1^ Processed soybean meal was a hydrolyzed soy protein product (HP 300) from Hamlet Protein (Findlay, OH, USA)

^2^ The trace mineral premix provided per kilogram of complete feed: 33 mg of Mn as Manganous oxide, 110 mg of Fe as ferrous sulfate, 110 mg of Zn as zinc sulfate, 16.5 mg of Cu as copper sulfate, 0.30 mg of I as ethylenediamine dihydroiodide, and 0.30 mg of Se as sodium selenite

^3^ The vitamin premix provided per kilogram of complete feed: 6,614 IU of vitamin A as vitamin A acetate, 992 IU of vitamin D_3_, 19.8 IU of vitamin E, 2.64 mg of vitamin K as menadione sodium bisulfate, 0.03 mg of vitamin B_12_, 4.63 mg of riboflavin, 18.52 mg of D-pantothenic acid as calcium pantothenate, 24.96 mg of niacin, and 0.07 mg of biotin

^4^ *ME* Metabolizable energy

^5^ *SID* Standardized ileal digestible

^6^ *ATTD* Apparent total tract digestible

### Sample and data collection

On each of d 20 and 42, 24 pigs (*n* = 12 per treatment on each day) were euthanized by the penetration of a captive bolt followed by exsanguination. The euthanized pigs on d 20 were randomly selected within the same block by initial BW of each sex. After euthanasia, jejunal mucosa, jejunal tissues, and jejunal digesta were collected at d 20 and 42. Jejunum tissues were obtained from 3 to 4 m after the pyloric valve of stomach of pigs. The jejunal tissues (20 cm) were flushed with 0.9% saline solution to remove jejunal digesta and the flushed jejunal tissues were collected. The first 15 cm was used to collect jejunal mucosa by scraping the mucosa layer in the jejunum using a glass microscope slide and the remaining 5 cm was fixed in 10% buffered formaldehyde to be used for Ki-67^+^ staining and histological evaluation [8, 23]. Jejunal mucosa were collected for tumor necrosis factor-alpha (TNF-α), interleukin-8 (IL-8), immunoglobulin A (IgA), and immunoglobulin G (IgG) as indicators of immune response status and protein carbonyl and malondialdehyde (MDA) as oxidative damage products. Jejunal digesta samples were collected into 15-mL tubes, kept on ice, and viscosity was measured on the sampling date, after collection. The jejunal mucosa samples were transferred to the freezer at −80 °C for further process and analysis including DNA extraction, immune responses, and oxidative damage products.

### Viscosity of jejunal digesta

Viscosity of jejunal digesta was measured using a viscometer (Brookfield Digital Viscometer, Model DV-II Version 2.0, Brookfield Engineering Laboratories Inc., Stoughton, MA, USA) following the previous studies [24, 25]. The 15-mL tubes containing jejunal digesta were centrifuged at 1,000 × *g* at 4 °C for 10 min to obtain the liquid phase for supernatant. After the first centrifuging process, the liquid phase was transferred to a 2-mL tube to centrifuge at 10,000 × *g* at 4 °C for 10 min. The supernatant was transferred to another 2-mL tube for further measurement. The 0.5 mL of centrifuged jejunal digesta were placed in the viscometer set at 25 °C. Viscosity measurement was the average between 45.0/s and 22.5/s shear rates, and the viscosity were recorded as apparent viscosity in millipascal seconds (mPa·s). The viscosity was measured 3 times per jejunal digesta sample with 2 internal replications [23, 25].

### Microbial diversity and relative abundance of mucosa-associated microbiota in the jejunum

The jejunal mucosa samples were sent to Zymo Research Corporation (Irvine, CA, USA) to determine microbial diversity and relative abundance (RA) of mucosa-associated microbiota in the jejunum [10, 26]. Jejunal mucosa samples were used for DNA extraction for 16S rRNA sequencing using the ZymoBIOMICS-96 MagBead DNA kit (Zymo Research). The extracted DNA samples were prepared for targeted sequencing with the Quick-16S Primer Set V3–V4 (Zymo Research) and NGS Library Preparation Kit for microbial analysis. These primers were custom-designed by Zymo Research to provide the best coverage of the 16S gene. The final PCR products were quantified with qPCR fluorescence readings and pooled together based on equal molarity. The final pooled library was cleaned up with the Select-a-Size DNA Clean & Concentrator (Zymo Research), then quantified with TapeStation (Agilent Technologies, Santa Clara, CA, USA) and Qubit (Thermo Fisher Scientific, Waltham, WA, USA). For sequencing, the final Library was sequenced on Illumina NextSeq 2000 (Illumina, San Diego, CA, USA) with a p1 (cat 20075294) reagent kit (600 cycles). The sequencing was performed with 30% PhiX spike-in using the Phix Control kit V3. Unique amplicon sequences were inferred from raw reads using the DADA2 pipeline [27]. Chimeric sequences were also removed with the DADA2 pipeline. The depth of sequencing coverage was > 20,000 × sample. Taxonomy was assigned with the Greengenes and Silva database, as references. Alpha diversity (Chao 1, Shannon, and Simpson indices) was evaluated with MicrobiomeAnalyst (QC, CA) [10, 28]. The ASV data were transformed to RA for further statistical analysis, and the ASV data with less than 0.50% abundance within each level were combined as “others”.

### Immune responses and oxidative damage products in the jejunum

Jejunal mucosa samples (0.5 g) were weighed and ground using a homogenizer (Tissuemiser, Thermo Fisher Scientific Inc., Rockford, IL, USA) on ice in 1 mL phosphate-buffered saline for 30 s. The homogenate was centrifuged at 14,000 × *g*, at 4 °C for 30 min to obtain supernatant, which was used to determine the contents of total protein, IgA, IgG, TNF-α, IL-8, IL-6, protein carbonyl, and MDA. The supernatant was pipetted off and kept at −80 °C. The content of total protein of mucosa was determined using the kit Pierce BCA Protein Assay (23225#, Thermo Fisher Scientific Inc.) to calculate the contents of IgA, IgG, TNF-α, IL-8, IL-6, protein carbonyl, and MDA per milligram of protein in the jejunal mucosa sample. The mucosa samples were diluted to 1:32 with distilled water to analyze total protein of mucosa. The contents of IgA and IgG were analyzed using an ELISA kit for pig IgA (E101-102, Bethyl Laboratories, Inc., Montgomery, TX, USA) and pig IgG (E101-104, Bethyl Laboratories, Inc.), respectively. The mucosa samples were diluted to 1:1,000 and 1:1,600 with PBS to analyze IgA and IgG, respectively. The contents of MDA and protein carbonyl were measured by commercial kits (Cell Biolabs, Inc., San Diego, CA, USA) following the protocols of the manufacturer. The contents of TNF-α, IL-8, and IL-6 in jejunal mucosa were measured by ELISA kits (R&D Systems, Minneapolis, MN, USA) following Deng et al. [12].

### Intestinal morphology and crypt cell proliferation in the jejunum

**Fig. 1:**
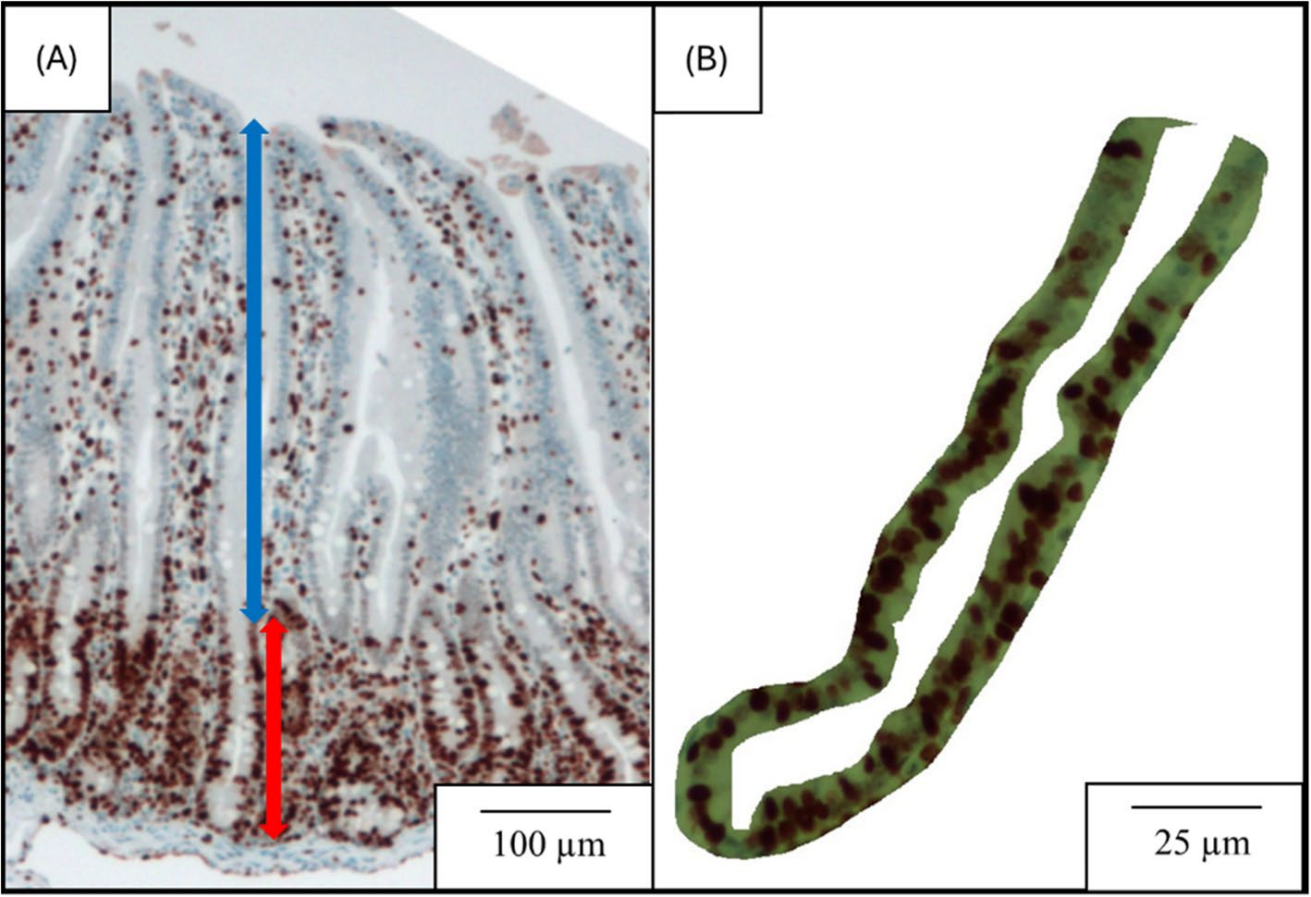
Representative images for the determination of intestinal morphology and crypt cell proliferation were taken from mounted slides after immunohistochemical staining with Ki-67. **A** Fifteen images at 40 × magnification showing clearly visible and well-oriented villi and their associated crypts were obtained for each sample to measure villus height (from the top to the base of the villus, as indicated with the double arrow blue line) and crypt depth (from the base of the villus to the bottom of the crypt, as indicated with a double arrow red line). **B** Fifteen images at 100 × magnification of the crypts were captured for each sample to determine the proportion and count of Ki-67^+^ cells in the jejunal crypt as an indicator of enterocyte proliferation

After 48 h in 10% buffered formaldehyde solution, 2 sections of the jejunum per pig were transversely cut, placed into a cassette in 70% ethanol, and sent to the University of North Carolina Histology Laboratory (UNC School of Medicine, Chapel Hill, NC, USA) for dehydration, embedment, and Ki-67^+^ immunohistochemistry staining for morphological evaluation and to evaluate cell proliferation in the crypt following previous studies [25, 26]. Pictures of villi and crypts were taken at 40× magnification using a camera Infinity 2–2 digital CCD attached to a microscope Olympus CX31 (Lumenera Corporation, Ottawa, Canada) for intestinal morphology, and the villus height (VH) and crypt depth (CD) were measured. The VH to CD ratio (VH:CD) was also determined. Pictures of crypts in 100 × magnification were taken for Ki-67^+^ cell measurement. The ImageJS software was used for calculating the percentage of dyed Ki-67^+^ cells in the total cells in the jejunal crypt. The proportion and count of Ki-67^+^ cells were used as an indicator of enterocyte proliferation in the jejunal crypt [10, 26]. All analyses of morphology were executed by the same person, and the average 15 measurements of each sample were calculated and reported as one number per sample (Fig. 1).

### Chemical analyses

Experimental diets were finely ground and dried in the forced-air drying oven at 135 °C for 2 h to determine dry matter (DM; method 930.15) and ether extract (EE) was analyzed using anhydrous diethyl ether (method 920.39) as described in AOAC [29]. Nitrogen content in diets was measured using a Truspec N Nitrogen Determinator (LECO Corp., St. Joseph, MI, USA) to determine crude protein (CP; 6.25 × nitrogen). Experimental diets were analyzed for gross energy (GE) using bomb calorimetry (Parr 1261, Parr Instrument Co., Moline, IL, USA), detecting energy released during the complete combustion of a sample. The diets were analyzed for neutral detergent fiber (NDF; method 2002.04) and acid detergent fiber (ADF; method 973.18) as described in AOAC [29], using an ANKOM 200 Fiber Analyzer (ANKOM Technology Corp., Macedon, NY, USA).

### Statistical analyses

Experimental data were analyzed using the MIXED procedure in SAS 9.4 (SAS Inst., Cary, NC, USA). The statistical model included dietary SBM levels, days post-weaning, and interaction between dietary SBM levels (low and high SBM diets) and days post-weaning (20 d and 42 d post-weaning) as fixed effects. Initial BW (light and heavy) and sex (male and female) were included as random effects. The experimental unit was a pen. The least squares mean of each treatment was calculated. The statistical significance and tendency were declared at *P* < 0.05 and 0.05 ≤ *P* < 0.10, respectively.

## Results

### Viscosity of jejunal digesta

**Fig. 2:**
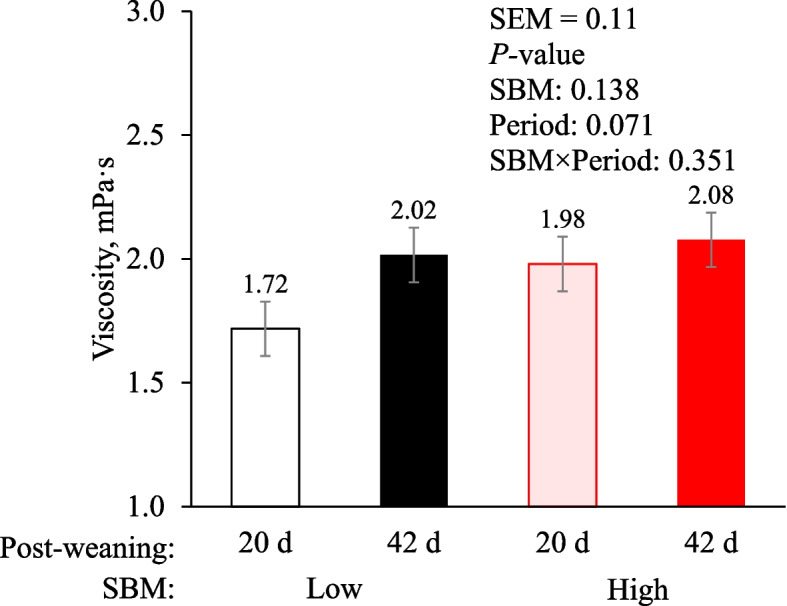
Jejunal digesta viscosity (mPa·s) of nursery pigs fed low or high soybean meal (SBM) diets at different days post-weaning (20 d and 42 d post-weaning). Experimental unit was a pig and each treatment had 12 replicates

Pigs tended to have lower (*P* = 0.071) viscosity of jejunal digesta at 20 d post-weaning than at 42 d post-weaning (Fig. 2). However, there were no differences in viscosity of jejunal digesta by dietary SBM levels and the interaction between dietary SBM levels and days post-weaning.

### Microbial diversity and relative abundance of mucosa-associated microbiota in the jejunum

**Fig. 3:**
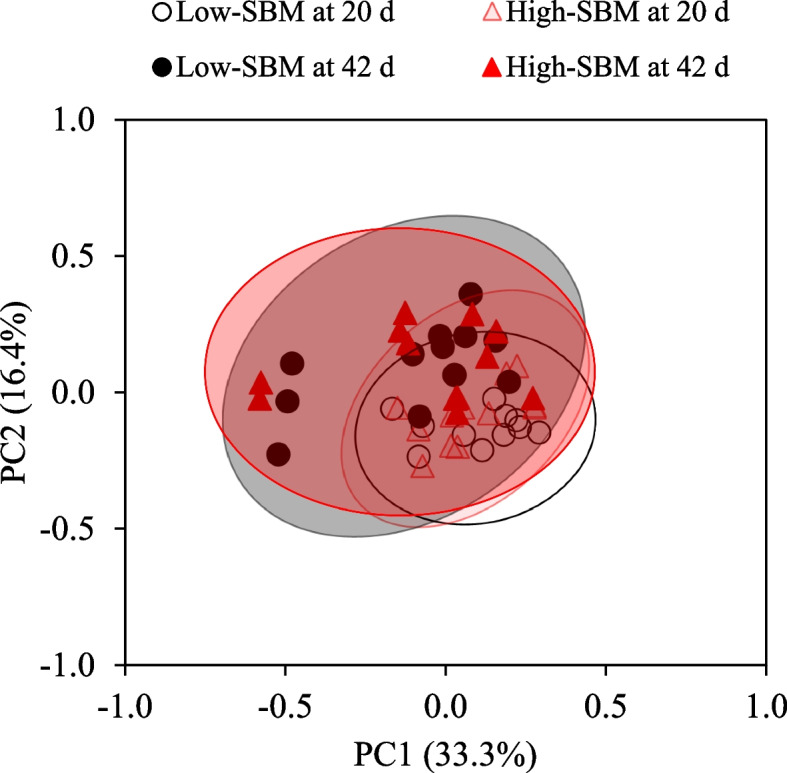
Principal coordinate analysis (PCoA) plot in the jejunal mucosa-associated microbiota at the genus level of nursery pigs fed low or high soybean meal (SBM) diets at different days post-weaning (20 d and 42 d post-weaning). The *X*-axis and *Y*-axis represent the principal coordinates axes, with the percentages indicating the proportion of variation explained by each coordinate. Points of different colors correspond to samples from different treatments, and the closer 2 points are, the more similar their genus composition. The Bray–Curtis distance analysis showed no difference between pigs fed low and high SBM diets (*P* = 0.645), but showed difference between 20 and 42 d post-weaning (*P* < 0.01). Experimental unit was a pen, and each treatment had 12 replicates

Pigs had lower (*P* < 0.05) Chao1, Shannon, and Simpson indices in the jejunal mucosa at 20 d post-weaning than 42 d post-weaning (Table 2). Pigs at 20 d post-weaning and 42 d post-weaning showed a difference (*P* < 0.01) in beta diversity, but low and high SBM diets did not differ in beta diversity (Fig. 3). There were no differences in Chao1, Shannon, and Simpson indices in the jejunal mucosa by dietary SBM levels and the interaction between dietary SBM levels and days post-weaning.

**Table 2:** Alpha diversity of jejunal mucosa-associated microbiota at the genus level in nursery pigs fed low or high soybean meal (SBM) diets at different days post-weaning^1^

SBM:	Low		High			*P-*value		
Post-weaning:	20 d	42 d	20 d	42 d	SEM^2^	SBM	Period	SBM × Period
Chao1	35.9	53.6	41.6	51.8	3.5	0.586	< 0.001	0.289
Shannon	1.54	2.09	1.69	2.02	0.12	0.747	< 0.001	0.377
Simpson	0.63	0.77	0.68	0.75	0.03	0.623	0.003	0.326

^1^ Experimental unit was a pig and each treatment had 12 replicates

^2^ *SEM* Standard error of the mean

Pigs fed high SBM diets had lower (*P* < 0.05) RA of Firmicutes in the jejunal mucosa than pigs fed low SBM diets (Table 3). Pigs had greater (*P* < 0.05) RA of Firmicutes and lower (*P* < 0.05) RA of Actinobacteria and Euryarchaeota in the jejunal mucosa at 20 d post-weaning than at 42 d post-weaning.

**Table 3:** Relative abundance (%) of jejunal mucosa-associated microbiota at the phylum and family level in nursery pigs fed low or high soybean meal (SBM) diets at different days post-weaning^1^

SBM:	Low		High		SEM^2^	*P-*value		
Post-weaning:	20 d	42 d	20 d	42 d		SBM	Period	SBM × Period
Phylum								
Firmicutes	76.52	63.53	64.82	58.62	4.01	0.038	0.018	0.387
Actinobacteria	12.40	21.37	17.77	24.41	3.54	0.227	0.028	0.736
Proteobacteria	8.74	9.61	11.21	10.82	4.68	0.664	0.955	0.882
Bacteroidetes	0.79	2.98	2.85	3.68	1.06	0.187	0.147	0.511
Euryarchaeota	0.19	1.18	0.27	1.35	0.26	0.607	< 0.001	0.857
Others	1.20	1.32	3.09	1.12	0.92	0.342	0.297	0.243
Family								
Lactobacillaceae	45.24	24.03	44.21	28.05	5.48	0.760	< 0.001	0.608
Bifidobacteriaceae	10.37	18.95	15.46	21.32	3.35	0.257	0.032	0.678
Helicobacteraceae	8.21	6.90	10.35	4.70	4.56	0.994	0.354	0.562
Lachnospiraceae	0.38	2.07	1.33	2.45	1.74	0.510	0.166	0.777
Ruminococcaceae	0.67	2.12	2.98	2.89	1.57	0.130	0.502	0.444
Clostridiales	< 0.01	3.26	< 0.01	< 0.01	3.50	0.099	0.077	0.963
Streptococcaceae	4.84	2.09	4.65	1.50	1.42	0.780	0.039	0.888
Veillonellaceae	3.05	3.27	3.10	2.75	0.76	0.747	0.929	0.699
Erysipelotrichaceae	1.52	3.91	1.19	3.92	1.01	0.863	0.007	0.851
Staphylococcaceae	5.10	9.00	3.79	5.35	2.48	0.075	0.050	0.394
Peptostreptococcaceae	10.78	2.60	1.36	1.89	4.61	0.179	0.308	0.247
Prevotellaceae	0.45	1.77	2.19	1.74	0.70	0.212	0.525	0.196
Coriobacteriaceae	1.67	1.53	1.77	2.34	0.44	0.285	0.612	0.402
Clostridiaceae	< 0.01	3.72	0.02	1.79	0.85	0.122	< 0.001	0.116
Leuconostocaceae	0.16	0.30	0.12	0.12	0.09	0.226	0.448	0.428
Xanthomonadaceae	2.15	2.82	2.18	6.17	2.16	0.217	0.092	0.226
Christensenellaceae	< 0.01	< 0.01	< 0.01	0.13	0.48	0.326	0.084	0.676
Methanobacteriaceae	0.19	1.13	0.24	1.32	0.25	0.611	< 0.001	0.776
Bacteroidales	< 0.01	0.30	0.12	0.51	0.31	0.255	0.043	0.943
Others	2.69	6.65	5.61	7.79	2.02	0.305	0.124	0.653

^1^ Experimental unit was a pig and each treatment had 12 replicates

^2^ *SEM* Standard error of the mean

Pigs fed high SBM diets tended to have lower (*P* = 0.075) RA of Staphylococcaceae in the jejunal mucosa than pigs fed low SBM diets. Pigs had greater (*P* < 0.05) RA of Lactobacillaceae and Streptococcaceae and lower (*P* < 0.05) RA of Bifidobacteriaceae, Erysipelotrichaceae, Clostridiaceae, Methanobacteriaceae, and Bacteroidales in the jejunal mucosa at 20 d post-weaning than at 42 d post-weaning.

Pigs fed high SBM diets had greater (*P* < 0.05) RA of *Prevotella* and tended to have lower (*P* = 0.077) RA of *Staphylococcus* in the jejunal mucosa than pigs fed low SBM diets (Table 4). Pigs had greater (*P* < 0.05) RA of *Lactobacillus* and *Streptococcus* and lower (*P* < 0.05) RA of *Bifidobacterium*, *Clostridiales*, *Lachnospiraceae*, and *Clostridium* in the jejunal mucosa at 20 d post-weaning than at d 42 post-weaning.

**Table 4:** Relative abundance (%) of jejunal mucosa-associated microbiota at the genus level in nursery pigs fed low or high soybean meal (SBM) diets at different days post-weaning^1^

SBM:	Low		High		SEM^2^	*P-*value		
Post-weaning:	20 d	42 d	20 d	42 d		SBM	Period	SBM × Period
*Lactobacillus*	45.21	24.02	44.21	28.05	5.48	0.757	0.001	0.610
*Bifidobacterium*	10.37	18.95	15.46	21.32	3.35	0.257	0.032	0.678
*Helicobacter*	8.21	6.90	10.35	4.70	4.56	0.994	0.354	0.562
*Clostridiales*	< 0.01	4.64	< 0.01	1.02	2.41	0.175	< 0.001	0.204
*Streptococcus*	4.80	2.08	4.65	1.50	1.41	0.790	0.038	0.876
*Ruminococcaceae*	0.17	1.20	1.60	1.91	1.13	0.134	0.345	0.612
*Staphylococcus*	5.07	8.93	3.76	5.32	2.47	0.077	0.052	0.400
*Lachnospiraceae*	< 0.01	0.45	< 0.01	1.03	0.69	0.172	0.007	0.918
*Clostridium*	< 0.01	3.72	0.02	1.79	0.85	0.122	< 0.001	0.116
*Romboutsia*	11.17	1.76	1.76	1.76	4.82	0.211	0.210	0.211
*Erysipelotrichaceae*	0.28	2.62	0.42	2.78	1.10	0.863	0.011	0.993
*Megasphaera*	0.95	1.53	1.56	1.21	0.45	0.685	0.759	0.214
*Olsenella*	1.13	1.04	1.19	1.89	0.38	0.224	0.414	0.295
*Prevotellaceae*	< 0.01	0.56	0.33	0.06	0.61	0.738	0.348	0.100
*Weissella*	0.08	0.33	0.16	0.15	0.09	0.459	0.082	0.068
*Prevotella*	0.18	0.45	1.14	0.94	0.36	0.046	0.911	0.502
*Stenotrophomonas*	2.24	2.90	2.19	6.25	2.17	0.227	0.087	0.215
*Mitsuokella*	0.57	0.99	0.84	0.70	0.26	0.965	0.576	0.258
*Subdoligranulum*	0.37	0.60	0.69	0.61	0.21	0.423	0.723	0.444
*Christensenellaceae*	< 0.01	< 0.01	< 0.01	0.13	0.48	0.326	0.084	0.676
*Sarcina*	3.30	< 0.01	< 0.01	< 0.01	1.60	0.278	0.278	0.277
Others	8.99	14.96	11.16	15.35	2.73	0.631	0.062	0.739

^1^ Experimental unit was a pig and each treatment had 12 replicates

^2^ *SEM* Standard error of the mean

Pigs fed high SBM diets had greater (*P* < 0.05) RA of *Prevotella copri* in the jejunal mucosa than pigs fed low SBM diets (Table 5). Pigs had greater (*P* < 0.05) RA of *Lactobacillus *spp*.*, *Bifidobacterium boum*, *Lactobacillus johnsonii*, *Lactobacillus vaginalis*, *Lactobacillus reuteri vaginalis*, and *Streptococcus orisratti* and lower (*P* < 0.05) RA of *Bifidobacterium dentium*, *Clostridium sp30555*, *Lactobacillus delbrueckii sp29223*, *Clostridium moniliforme*, and *Erysipelotrichaceae sp69504* in the jejunal mucosa at 20 d post-weaning than at 42 d post-weaning.

**Table 5:** Relative abundance (%) of jejunal mucosa-associated microbiota at the species level in nursery pigs fed low or high soybean meal (SBM) diets at different days post-weaning^1^

SBM:	Low		High		SEM^2^	*P-*value		
Post-weaning:	20 d	42 d	20 d	42 d		SBM	Period	SBM × Period
*Lactobacillus delbrueckii*	18.16	8.33	17.48	16.43	4.28	0.313	0.141	0.233
*Helicobacter rappini*	6.97	5.99	10.28	4.25	4.29	0.824	0.325	0.477
*Bifidobacterium thermophilum*	4.38	9.22	8.95	7.72	1.96	0.422	0.346	0.118
*Bifidobacterium dentium*	0.14	8.81	0.60	13.26	2.25	0.213	< 0.001	0.308
*Lactobacillus mucosae*	5.06	4.12	6.48	2.90	1.36	0.940	0.093	0.322
*Lactobacillus spp.*	7.71	1.09	7.32	0.29	1.37	0.629	< 0.001	0.868
*Bifidobacterium boum*	5.97	0.87	5.85	0.33	1.33	0.798	< 0.001	0.872
*Clostridiales sp30555*	< 0.01	4.79	< 0.01	1.14	2.35	0.164	< 0.001	0.198
*Lactobacillus delbrueckii (sp29223)*	0.99	3.65	2.03	3.25	1.06	0.727	0.038	0.429
*Lactobacillus sp29233*	1.62	5.23	1.69	2.67	1.44	0.317	0.069	0.290
*Lactobacillus johnsonii*	4.56	0.08	1.57	0.02	1.15	0.179	0.010	0.197
*Romboutsia ilealis*	11.17	1.76	1.76	1.76	4.82	0.211	0.210	0.211
*Clostridium moniliforme*	< 0.01	3.26	< 0.01	1.61	0.79	0.184	< 0.001	0.154
*Megasphaera sp36946*	0.94	1.50	1.55	1.17	0.44	0.706	0.802	0.201
*Helicobacter equorum*	1.29	0.87	< 0.01	0.42	0.67	0.189	0.998	0.527
*Erysipelotrichaceae sp69504*	0.12	2.17	0.06	2.33	1.14	0.957	0.020	0.902
*Lactobacillus vaginalis*	3.53	1.91	4.32	1.91	1.27	0.595	0.009	0.591
*Streptococcus parasuis (sp30071)*	1.37	0.47	1.77	0.44	0.57	0.738	0.050	0.700
*Lactobacillus equicursoris*	0.76	1.36	3.42	2.37	1.61	0.127	0.853	0.487
*Weissella thailandensis*	0.17	0.30	0.25	0.22	0.09	0.991	0.322	0.149
*Olsenella profusa*	0.77	0.40	0.71	1.00	0.39	0.372	0.898	0.286
*Stenotrophomonas maltophilia*	1.88	2.24	1.84	5.82	2.11	0.195	0.114	0.187
*Staphylococcus saprophyticus xylosus*	2.02	2.21	1.04	1.62	0.95	0.135	0.463	0.709
*Streptococcus hyointestinalis*	0.66	0.63	0.99	0.28	0.25	0.963	0.139	0.164
*Prevotella copri*	0.16	0.38	1.05	0.73	0.31	0.045	0.872	0.378
*Blautia wexlerae*	< 0.01	0.07	< 0.01	0.24	0.36	0.690	0.160	0.658
*Lactobacillus reuteri vaginalis*	1.24	0.06	0.60	0.01	0.24	0.133	< 0.001	0.205
*Streptococcus orisratti*	1.70	0.28	0.93	0.20	0.52	0.401	0.038	0.494
*Subdoligranulum sp35580 sp35585*	0.31	0.52	0.49	0.50	0.17	0.616	0.522	0.539
Others	22.86	31.08	23.42	28.79	4.93	0.847	0.135	0.750

^1^ Experimental unit was a pig and each treatment had 12 replicates

^2^ *SEM* Standard error of the mean

### Immune responses and oxidative damage products in the jejunum

Pigs fed high SBM diets tended to have greater (*P* = 0.091) IgA in the jejunal mucosa than pigs fed low SBM diets (Table 6). Pigs had greater (*P* < 0.05) IL-8 and protein carbonyl in the jejunal mucosa at 20 d post-weaning than at 42 d post-weaning. Pigs tended to have greater (*P* = 0.090) IgG in the jejunal mucosa at 20 d post-weaning than at 42 d post-weaning. Pigs tended to have lower (*P* = 0.059) IgA in the jejunal mucosa at 20 d post-weaning than at 42 d post-weaning. However, there were no differences in jejunal IL-6, TNF-α, and MDA by dietary SBM levels and the interaction between dietary SBM levels and days post-weaning.

**Table 6:** Immune responses and oxidative damage products in the jejunum of nursery pigs fed low or high soybean meal (SBM) diets at different days post-weaning^1^

SBM:	Low		High		SEM^2^	*P-*value		
Post-weaning:	20 d	42 d	20 d	42 d		SBM	Period	SBM × Period
Jejunum^3^, /mg of protein								
IgA, µg	3.03	3.28	3.21	4.32	0.35	0.091	0.059	0.226
IgG, µg	1.74	1.19	1.68	1.50	0.26	0.567	0.090	0.380
IL-8, ng	0.42	0.25	0.39	0.33	0.04	0.473	0.006	0.173
IL-6, pg	5.48	4.91	6.50	6.37	1.02	0.260	0.747	0.845
TNF-α, pg	0.70	0.72	0.96	0.70	0.14	0.186	0.177	0.117
Protein carbonyl, nmol	3.51	1.99	3.50	2.44	0.43	0.609	0.004	0.590
MDA, nmol	0.60	0.42	0.38	0.42	0.14	0.217	0.427	0.249

^1^ Experimental unit was a pig and each treatment had 12 replicates

^2^ *SEM* Standard error of the mean

^3^ *IgA* Immunoglobulin A, *IgG* Immunoglobulin G, *IL-8* Interleukin-8, *IL-6* Interleukin-6, *TNF-α* Tumor necrosis factor-alpha, *MDA* Malondialdehyde

### Intestinal morphology and crypt cell proliferation in the jejunum

Pigs fed high SBM diets had greater (*P* < 0.05) CD and tended to have lower (*P* = 0.064) VH:CD in the jejunum than pigs fed low SBM diets (Table 7). Pigs had greater (*P* < 0.05) proportion of Ki-67^+^ cells in the jejunal crypt at 20 d post-weaning than 42 d post-weaning. However, there were no differences in VH, count of Ki-67^+^ cells in the jejunal crypt by dietary SBM levels and the interaction between dietary SBM levels and days post-weaning.

**Table 7:** Intestinal morphology and crypt cell proliferation in the jejunum of nursery pigs fed low or high soybean meal (SBM) diets at different days post-weaning^1^

SBM:	Low		High		SEM^2^	*P-*value		
Post-weaning:	20 d	42 d	20 d	42 d		SBM	Period	SBM × Period
Villus height, µm	504	542	520	523	30	0.971	0.471	0.535
Crypt depth, µm	161	178	183	190	10	0.029	0.123	0.523
VH:CD	3.47	3.37	3.18	3.10	0.26	0.064	0.558	0.960
Ki-67^+^, %	37.3	30.7	38.2	31.1	2.3	0.668	< 0.001	0.846
Ki-67^+^, count	80.9	77.1	82.0	77.7	4.2	0.817	0.303	0.945

^1^ Experimental unit was a pig and each treatment had 12 replicates

^2^ *SEM* Standard error of the mean

## Discussion

Understanding the impact of anti-nutritional compounds in SBM on growth depression during the nursery phase is a key consideration for improving intestinal health and growth performance in pigs [1, 30]. In this study, pigs fed high SBM diets showed an increased RA of Staphylococcaceae in the jejunum. This is likely due to the presence of undigested proteins and NSP in SBM remaining in the intestine [12, 31], which may promote the growth of ammonia-producing and opportunistic pathogenic bacteria such as Staphylococcaceae [32]. *Staphylococcus*, a representative genus of opportunistic pathogens, includes *Staphylococcus aureus*, which is known to induce intestinal inflammation in nursery pigs [33]. Higher SBM inclusion levels are associated with increased contents of allergenic proteins such as β-conglycinin and glycinin, which are highly antigenic and resistant to digestion in the small intestine of pigs [34].

These undigested proteins may create a proteolytic environment that promotes the proliferation of Staphylococcaceae and stimulates immune responses in the intestinal mucosa of pigs [35]. In general, IgG functions to neutralize antigens and facilitate their clearance via opsonization [7, 36], whereas IgA plays a critical role in mucosal immunity by blocking antigen entry at mucosal surfaces such as the intestinal epithelium [37]. Previous studies reported that excessive SBM intake increases serum IgG levels, suggesting systemic immune activation [38], whereas reduced SBM inclusion did not increase serum IgG levels [39]. However, in this study no difference in jejunal IgG was observed, but increased jejunal IgA, potentially indicating that the immune response to SBM occurred primarily at the local mucosal level in the jejunum rather than systemically. In a previous study, IgA contents in the jejunal mucosa of nursery pigs increased as dietary glycinin levels increased from 0 to 8%, which is a major anti-nutritional compound in SBM [4]. The deviation between the previous studies was the site of immune response. Immunoglobulin A is an antibody that is abundantly present in the intestine, and in terms of intestinal immunity, IgA can be more likely to respond to dietary SBM compared to IgG [37]. Additionally, no differences were observed in pro-inflammatory cytokines such as IL-6 and TNF-α in response to dietary SBM, which reflect acute or systemic inflammatory responses. The increase in IgA observed in this study in response to dietary SBM may be due to enhanced T cell activation by glycinin in SBM, as indicated by an increased CD4^+^/CD8^+^ ratio [4]. This shift in T cell populations suggests a polarization toward the Th2 pathway, which is primarily responsible for IL-4 production [40]. Interleukin-4 plays a critical role in promoting B cell class switching to IgA-producing cells [4, 41], and may represent a potential mechanism underlying the mucosal immune response for IgA to dietary SBM in this study. Therefore, based on the results of this and previous studies, the increased RA of Staphylococcaceae and increased jejunal IgA suggest that dietary SBM may influence mucosal immunity either directly, through antigenic stimulation, or indirectly, by altering the intestinal microbiota, which subsequently affects immune activation in the jejunum of nursery pigs. In addition, NSP content in SBM can negatively affect intestinal health [25]. Within the NSP fraction, β-mannan is highly abundant in SBM and may negatively affect intestinal health in pigs by increasing viscosity due to its soluble form [25] as well as through its structural similarities to outer cell wall of pathogenic bacteria [42]. In this study, the RA of *Prevotella* increased in response to high SBM diets with increased NSP content, which is consistent with the previous findings [25, 43]. This increase may be attributed to increased NSP content in SBM, which serves as a fermentable substrate for *Prevotella* growth.

This study also showed that SBM negatively affects intestinal morphology. A reduction in villus height reflects a decrease in the number of mature absorptive epithelial cells, whereas increase in CD indicates increased cellular turnover or potential mucosal damage [44]. Such structural changes are directly linked to reduced nutrient absorption, and the VH:CD is widely used as a representative marker of intestinal health [13, 45]. Soybean meal contains heat-stable anti-nutritional compounds, among which glycinin and β-conglycinin are the major antigenic proteins [1]. These proteins can transiently increase crypt cell proliferation, leading to villus atrophy and malabsorption in pigs [1, 46]. In this study, however, the proportion and count of cells proliferating in the crypt were not increased by the high dietary SBM diets, whereas the crypt depth in the jejunum was increased. A previous study reported that high SBM diets reduced the epithelial cell migration rate in the proximal jejunum [47], suggesting that even with unchanged cell turnover, crypt depth could increase due to reduced cell migration rate. In contrast, other previous studies reported that pigs fed high SBM diets showed reduced cell proliferation in the crypt [31]. These discrepancies may be due to differences in the post-weaning period [14, 48] and dietary SBM levels [25, 49].

The presence of soybean proteins in SBM has also been reported to increase the incidence of diarrhea in nursery pigs due to raffinose-family oligosaccharides [50]. These oligosaccharides are not digested in the small intestine, as pigs lack endogenous enzymes capable of hydrolyzing α-galactosidic linkages. As a result, they are heavily fermented in the large intestine, producing gas and causing diarrhea [7]. Therefore, the presence of SBM can induce immune responses, impair intestinal structure, and induce diarrhea, ultimately reducing intestinal health and growth in nursery pigs [35, 39].

After weaning, the number of opportunistic pathogens in the intestine increases, accompanied by a reduction in microbial diversity due to the abrupt transition to solid diets [51]. Lower microbial diversity indicates dysbiosis, which refers to an imbalance in the microbial community characterized by a reduction in beneficial microbiota or an increase in opportunistic pathogenic bacteria [52]. This suggests a high potential to be influenced by weaning stress, potentially increasing intestinal inflammation of nursery pigs [53]. In this study, pigs at 20 d post-weaning showed lower alpha diversity indices compared to those at 42 d post-weaning, indicating insufficient bacterial colonization and a vulnerable period during which intestinal health and intestinal immunity can be more compromised by external factors [53]. Interestingly, the RA of *Lactobacillus* was greater in pigs at 20 d post-weaning than in those at 42 d post-weaning. A possible reason for this is the high inclusion rate of whey permeate, which contains lactose, a preferential substrate that may promote the growth of *Lactobacillus* [54]. Even though whey permeate was provided as a source of lactose to supply energy, taking advantage of high lactase activity of young pigs and potentially mitigating the negative impacts of weaning stress during the early nursery phase, pigs at 20 d post-weaning still showed greater immune responses in the jejunum, including increased levels of IgG and IL-8, as well as greater oxidative damage products such as protein carbonyl, compared to those at 42 d post-weaning. A possible reason for this is that immaturity of the mucosal immune system, which lacks the regulatory capacity to modulate immune response efficiently [55]. Additionally, previous studies reported that pigs in the early nursery phase showed increased immune responses to pathogens on the epithelial cells compared to pigs in the later nursery phase [53, 56], indicating that the mucosal immune system is more susceptible to external factors during the early nursery phase. Thus, the findings of this study suggest that the early nursery phase in pigs is a critical and susceptible period for mucosal microbiota and intestinal health, likely due to intestinal immaturity, which leads to heightened pathogenic stimulation and increased local immune activation [16, 48]. This activation may also divert energy away from intestinal maintenance and growth toward immune defense.

An increase in proportion of Ki-67^+^ cells in the jejunal crypt was observed at 20 d post-weaning, indicating enhanced crypt cell proliferation. Ki-67 is an established marker of cellular proliferation and reflects regenerative response to epithelial injury [13, 23]. These findings suggest that the intestinal epithelium actively engages in regenerative processes to compensate for damage during this period. Therefore, based on the findings of this study, the immature digestive system during the early nursery phase likely limits the pigs’ ability to properly digest solid diet, leading to various negative impacts such as intestinal inflammation, structural damage to the intestine, which are closely related to growth retardation.

Nursery pigs showed greater jejunal inflammation and intestinal damage, as indicated by increased immune responses, oxidative damage, and crypt cell proliferation at 20 d post-weaning than at 42 d post-weaning. However, the negative impacts of high SBM diets on jejunal inflammation and intestinal damage were consistent compared to low SBM diets at both 20 d and 42 d post-weaning, indicating no interaction between dietary SBM levels and days post-weaning period. This may be because pigs had adapted to the dietary SBM by 20 d post-weaning, which could have attenuated the immune response in the jejunum, as previous studies suggest that pigs start adapting to dietary SBM within 7 to 10 d after introduction to the diet [19]. In addition, the immune system becomes partially developed by around 1 month of age [57], which corresponds to 20 d post-weaning, as observed in this study. These factors may explain why the high-SBM diet showed similar impacts on intestinal health at 20 d and 42 d post-weaning.

## Conclusion

Nursery pigs fed high SBM diets showed greater RA of *Staphylococcus*, a greater immune response, and a decreased VH:CD in the jejunum compared to pigs fed low SBM diets. In addition, pigs at 20 d post-weaning were more susceptible to jejunal inflammation and intestinal damage than pigs at 42 d post-weaning, as indicated by lower microbial diversity and increased RA of *Streptococcus*, increased immune responses, oxidative damage, and crypt cell proliferation in the jejunum. However, the negative impacts of high SBM diets on jejunal inflammation and intestinal damage were consistent compared to low SBM diets at 20 d and 42 d post-weaning. This suggests that the negative impacts of dietary SBM on the jejunal immune response and intestinal health during the early nursery phase may have been mitigated by partial adaptation to dietary SBM established by 20 days post-weaning, resulting in similar impacts during both the early and later nursery phases. 

## Supplementary Information

Additional file 1: Table S1 Fecal score of nursery pigs fed low or high soybean meal diets. Table S2 Growth performance of nursery pigs fed low or high soybean meal diets.

## Data Availability

All data generated or analyzed during this study are available from the corresponding author upon reasonable request.

## Abbreviations

ADF: Acid detergent fiber
ATTD: Apparent total tract digestible
BW: Body weight
Ca: Calcium
CD: Crypt depth
CP: Crude protein
DM: Dry matter
EE: Ether extract
GE: Gross energy
IgA: Immunoglobulin A
IgG: Immunoglobulin G
IL-6: Interleukin-6
IL-8: Interleukin-8
MDA: Malondialdehyde
ME: Metabolizable energy
NDF: Neutral detergent fiber
NSP: Non-starch polysaccharides
P: Phosphorus
RA: Relative abundance
SBM: Soybean meal
SID: Standardized ileal digestible
TNF-α: Tumor necrosis factor-alpha
VH: Villus height
VH:CD: Villus height to crypt depth ratio

## References

[1] Deng Z, Kim SW. Opportunities and challenges of soy proteins with different processing applications. Antioxidants. 2024;13:569. 10.3390/antiox13050569. 38790674 10.3390/antiox13050569 PMC11117726

[2] Fan MZ, Sauer WC, De Lange CFM. Amino acid digestibility in soybean meal, extruded soybean and full-fat canola for early-weaned pigs. Anim Feed Sci Technol. 1995;52:189–203. 10.1016/0377-8401(94)00732-O.

[3] Choi H, You SJ, Kim BG. Amino acid supplementation during the adaptation period did not affect the standardized ileal digestibility of amino acids in corn and soybean meal fed to pigs. Anim Biosci. 2023;37:492–9. 10.5713/ab.23.0331. 37946417 10.5713/ab.23.0331 PMC10915196

[4] Sun P, Li D, Dong B, Qiao S, Ma X. Effects of soybean glycinin on performance and immune function in early weaned pigs. Arch Anim Nutr. 2008;62:313–21. 10.1080/17450390802066419. 18763625 10.1080/17450390802066419

[5] Kim SW, Knabe DA, Hong KJ, Easter RA. Use of carbohydrases in corn–soybean meal-based nursery diets. J Anim Sci. 2003;81:2496–504. 10.2527/2003.81102496x. 14552377 10.2527/2003.81102496x

[6] Deng Z, Choi H, Kim SW. Impacts of replacing soybean meal with processed soybean meal on intestinal health and growth of nursery pigs challenged with F18^+^*Escherichia coli*. Anim Biosci. 2025;38:728–38. 10.5713/ab.24.0566. 39483001 10.5713/ab.24.0566 PMC11917416

[7] Ma X, Shang Q, Hu J, Liu H, Brøkner C, Piao X. Effects of replacing soybean meal, soy protein concentrate, fermented soybean meal or fish meal with enzyme-treated soybean meal on growth performance, nutrient digestibility, antioxidant capacity, immunity and intestinal morphology in weaned pigs. Livest Sci. 2019;225:39–46. 10.1016/j.livsci.2019.04.016.

[8] Deng Z, Duarte ME, Kim SW. Efficacy of soy protein concentrate replacing animal protein supplements in mucosa-associated microbiota, intestinal health, and growth performance of nursery pigs. Anim Nutr. 2023;14:235–48. 10.1016/j.aninu.2023.06.007. 37600837 10.1016/j.aninu.2023.06.007 PMC10432921

[9] Arpaia N, Campbell C, Fan X, Dikiy S, Van Der Veeken J, Deroos P, et al. Metabolites produced by commensal bacteria promote peripheral regulatory T-cell generation. Nature. 2013;504:451–5. 10.1038/nature12726. 24226773 10.1038/nature12726 PMC3869884

[10] Gormley AR, Duarte ME, Deng Z, Kim SW. *Saccharomyces* yeast postbiotics mitigate mucosal damages from F18^+^ *Escherichia coli* challenges by positively balancing the mucosal microbiota in the jejunum of young pigs. Anim Microbiome. 2024;6:73. 10.1186/s42523-024-00363-y. 39707576 10.1186/s42523-024-00363-y PMC11662450

[11] Duarte ME, Kim SW. Significance of mucosa-associated microbiota and its impacts on intestinal health of pigs challenged with F18^+^*E. coli*. Pathogens. 2022;11:589. 10.3390/pathogens11050589. 35631110 10.3390/pathogens11050589 PMC9145386

[12] Deng Z, Duarte ME, Kim SY, Hwang Y, Kim SW. Comparative effects of soy protein concentrate, enzyme-treated soybean meal, and fermented soybean meal replacing animal protein supplements in feeds on growth performance and intestinal health of nursery pigs. J Anim Sci Biotechnol. 2023;14:89. 10.1186/s40104-023-00888-3. 37393326 10.1186/s40104-023-00888-3 PMC10315043

[13] Kim SW, Duarte ME. Understanding intestinal health in nursery pigs and the relevant nutritional strategies. Anim Biosci. 2021;34:338–44. 10.5713/ab.21.0010. 33705620 10.5713/ab.21.0010 PMC7961202

[14] Moeser AJ, Pohl CS, Rajput M. Weaning stress and gastrointestinal barrier development: implications for lifelong gut health in pigs. Anim Nutr. 2017;3:313–21. 10.1016/j.aninu.2017.06.003. 29767141 10.1016/j.aninu.2017.06.003 PMC5941262

[15] Li P, Niu Q, Wei Q, Zhang Y, Ma X, Kim SW, et al. Microbial shifts in the porcine distal gut in response to diets supplemented with *Enterococcus**faecalis* as alternatives to antibiotics. Sci Rep. 2017;7:41395. 10.1038/srep41395. 28165001 10.1038/srep41395 PMC5292720

[16] Duarte ME, Kim SW. Intestinal microbiota and its interaction to intestinal health in nursery pigs. Anim Nutr. 2022;8:169–84. 10.1016/j.aninu.2021.05.001. 34977387 10.1016/j.aninu.2021.05.001 PMC8683651

[17] Smith F, Clark JE, Overman BL, Tozel CC, Huang JH, Rivier JEF, et al. Early weaning stress impairs development of mucosal barrier function in the porcine intestine. Am J Physiol Gastrointest Liver Physiol. 2010;298:G352–63. 10.1152/ajpgi.00081.2009. 19926814 10.1152/ajpgi.00081.2009 PMC2838512

[18] Friesen KG, Goodband RD, Nelssen JL, Blecha F, Reddy DN, Reddy PG, et al. The effect of pre-and postweaning exposure to soybean meal on growth performance and on the immune response in the early-weaned pig. J Anim Sci. 1993;71:2089–98. 10.2527/1993.7182089x. 8376233 10.2527/1993.7182089x

[19] Barratt ME, Strachan PJ, Porter P. Antibody mechanisms implicated in digestive disturbances following ingestion of soya protein in calves and piglets. Clin Exp Immunol. 1978;31:305. 565686 PMC1541224

[20] Skinner LD, Levesque CL, Wey D, Rudar M, Zhu J, Hooda S, et al. Impact of nursery feeding program on subsequent growth performance, carcass quality, meat quality, and physical and chemical body composition of growing-finishing pigs. J Anim Sci. 2014;92:1044–54. 10.2527/jas.2013-6743. 24492546 10.2527/jas.2013-6743

[21] NRC. Nutrient requirements of swine*.* 11th ed. Washington, DC, USA: National Academies Press; 2012.

[22] Cowieson AJ, Ruckebusch J-P, Sorbara JOB, Wilson JW, Guggenbuhl P, Tanadini L, et al. A systematic view on the effect of microbial phytase on ileal amino acid digestibility in pigs. Anim Feed Sci Technol. 2017;231:138–49. 10.1016/j.anifeedsci.2017.07.007.

[23] Choi H, Duarte YG, Pasquali GAM, Kim SW. Investigation of the nutritional and functional roles of a combinational use of xylanase and β-glucanase on intestinal health and growth of nursery pigs. J Anim Sci Biotechnol. 2024;15:63. 10.1186/s40104-024-01021-8. 38704593 10.1186/s40104-024-01021-8 PMC11070102

[24] Duarte ME, Zhou FX, Dutra WM Jr, Kim SW. Dietary supplementation of xylanase and protease on growth performance, digesta viscosity, nutrient digestibility, immune and oxidative stress status, and gut health of newly weaned pigs. Anim Nutr. 2019;5:351–8. 10.1016/j.aninu.2019.04.005. 31890911 10.1016/j.aninu.2019.04.005 PMC6920460

[25] Baker JT, Deng Z, Sokale A, Frederick B, Kim SW. Nutritional and functional roles of β-mannanase on intestinal health and growth of newly weaned pigs fed two different types of feeds. J Anim Sci. 2024;102:skae206. 10.1093/jas/skae206. 39044687 10.1093/jas/skae206 PMC11306790

[26] Duarte ME, Deng Z, Kim SW. Effects of dietary *Lactobacillus* postbiotics and bacitracin on the modulation of mucosa-associated microbiota and pattern recognition receptors affecting immunocompetence of jejunal mucosa in pigs challenged with enterotoxigenic F18^+^ *Escherichia coli*. J Anim Sci Biotechnol. 2024;15:139. 10.1186/s40104-024-01098-1. 39390608 10.1186/s40104-024-01098-1 PMC11468193

[27] Callahan BJ, McMurdie PJ, Rosen MJ, Han AW, Johnson AJA, Holmes SP. DADA2: high-resolution sample inference from Illumina amplicon data. Nat Methods. 2016;13:581–3. 10.1038/nmeth.3869. 27214047 10.1038/nmeth.3869 PMC4927377

[28] Choi H, Rocha GC, Kim SW. Effects of dietary supplementation of myristic acid on jejunal mucosa-associated microbiota, mucosal immunity, and growth performance of nursery pigs. Anim Sci J. 2025;96:e70027. 10.1111/asj.70027. 39777830 10.1111/asj.70027 PMC11707569

[29] AOAC. Official Methods of Analysis of AOAC International. 18th ed. Gaithersburg, MD: AOAC International; 2005.

[30] Kim SW, Van Heugten E, Ji F, Lee CH, Mateo RD. Fermented soybean meal as a vegetable protein source for nursery pigs: I. effects on growth performance of nursery pigs. J Anim Sci. 2010;88:214–24. 10.2527/jas.2009-1993. 19783703 10.2527/jas.2009-1993

[31] Zhao Y, Qin G, Sun Z, Zhang X, Bao N, Wang T, et al. Disappearance of immunoreactive glycinin and β-conglycinin in the digestive tract of piglets. Arch Anim Nutr. 2008;62:322–30. 10.1080/17450390802190318. 18763626 10.1080/17450390802190318

[32] Tan K, Bian Z, Liang H, Hu W, Xia M, Han S, et al. Enzymolytic soybean meal—impact on growth performance, nutrient digestibility, antioxidative capacity, and intestinal health of weaned piglets. Front Vet Sci. 2024;11:1381823. 10.3389/fvets.2024.1381823. 38585301 10.3389/fvets.2024.1381823 PMC10995376

[33] Verstappen KM, Willems E, Fluit AC, Duim B, Martens M, Wagenaar JA. *Staphylococcus* aureus nasal colonization differs among pig lineages and is associated with the presence of other staphylococcal species. Front Vet Sci. 2017;4:97. 10.3389/fvets.2017.00097. 28691012 10.3389/fvets.2017.00097 PMC5481302

[34] Park S, Lee JW, Cowieson AJ, Pappenberger G, Woyengo TA. Soybean meal allergenic protein degradation and gut health of piglets fed protease-supplemented diets. J Anim Sci. 2020;98:skaa308. 10.1093/jas/skaf101. 10.1093/jas/skaa308 PMC7568435 32927480

[35] Song YS, Pérez VG, Pettigrew JE, Martinez-Villaluenga C, de Mejia EG. Fermentation of soybean meal and its inclusion in diets for newly weaned pigs reduced diarrhea and measures of immunoreactivity in the plasma. Anim Feed Sci Technol. 2010;159:41–9. 10.1016/j.anifeedsci.2010.04.011.

[36] Li DF, Nelssen JL, Reddy PG, Blecha F, Klemm RD, Giesting DW, et al. Measuring suitability of soybean products for early-weaned pigs with immunological criteria. J Anim Sci. 1991;69:3299–307. 10.2527/1991.6983299x. 1894566 10.2527/1991.6983299x

[37] Chen K, Magri G, Grasset EK, Cerutti A. Rethinking mucosal antibody responses: IgM, IgG and IgD join IgA. Nat Rev Immunol. 2020;20:427–41. 10.1038/s41577-019-0261-1. 32015473 10.1038/s41577-019-0261-1 PMC10262260

[38] Dierick N, Decuypere J, Molly K, Vanderbeke E. Microbial protease addition to a soybean meal diet for weaned piglets: effects on performance, digestion, gut flora and gut function. In: Muzquiz M, Hill GD, Burbano C, Cuadrado C, Pedrosa MM, editors. Recent advances of research in antinutritional factors in legume seeds and oilseeds. Wageningen: Academic; 2004. p. 229–33.

[39] Li DF, Nelssen JL, Reddy PG, Blecha F, Hancock JD, Allee GL, et al. Transient hypersensitivity to soybean meal in the early-weaned pig. J Anim Sci. 1990;68:1790–9. 10.2527/1990.6861790x. 2384373 10.2527/1990.6861790x

[40] Mosmann TR, Coffman RL. TH1 and TH2 cells: different patterns of lymphokine secretion lead to different functional properties. Annu Rev Immunol. 1989;7:145–73. 10.1146/annurev.iy.07.040189.001045. 2523712 10.1146/annurev.iy.07.040189.001045

[41] Cerutti A. The regulation of IgA class switching. Nat Rev Immunol. 2008;8:421–34. 10.1038/nri2322. 18483500 10.1038/nri2322 PMC3062538

[42] Kiarie EG, Steelman S, Martinez M. Does supplementing β-mannanase modulate the feed-induced immune response and gastrointestinal ecology in poultry and pigs? An appraisal. Front Anim Sci. 2022;3:875095. 10.3389/fanim.2022.875095.

[43] Amat S, Lantz H, Munyaka PM, Willing BP. *Prevotella* in pigs: the positive and negative associations with production and health. Microorganisms. 2020;8:1584. 10.3390/microorganisms8101584. 33066697 10.3390/microorganisms8101584 PMC7602465

[44] Kai Y. Intestinal villus structure contributes to even shedding of epithelial cells. Biophys J. 2021;120:699–710. 10.1016/j.bpj.2021.01.003. 33453270 10.1016/j.bpj.2021.01.003 PMC7896031

[45] Pluske JR, Williams IH, Aherne FX. Maintenance of villous height and crypt depth in piglets by providing continuous nutrition after weaning. Anim Sci. 1996;62:131–44. 10.1017/S1357729800014417.

[46] Zhou SF, Sun ZW, Ma LZ, Yu JY, Ma CS, Ru YJ. Effect of feeding enzymolytic soybean meal on performance, digestion and immunity of weaned pigs. Asian-Australas J Anim Sci. 2010;24:103–9. 10.5713/ajas.2011.10205.

[47] Qiao S, Li D, Jiang J, Zhou H, Li J, Thacker PA. Effects of moist extruded full-fat soybeans on gut morphology and mucosal cell turnover time of weanling pigs. Asian-Australas J Anim Sci. 2003;16:63–9. 10.5713/ajas.2003.63.

[48] Salak-Johnson JL, Webb SR. Short-and long-term effects of weaning age on pig innate immune status. Open J Anim Sci. 2018;8:137–50. 10.4236/ojas.2018.82010.

[49] Yuan L, Chang J, Yin Q, Lu M, Di Y, Wang P, et al. Fermented soybean meal improves the growth performance, nutrient digestibility, and microbial flora in piglets. Anim Nutr. 2017;3:19–24. 10.1016/j.aninu.2016.11.003. 29767125 10.1016/j.aninu.2016.11.003 PMC5941061

[50] Liying Z, Li D, Qiao S, Johnson EW, Li B, Thacker PA, et al. Effects of stachyose on performance, diarrhoea incidence and intestinal bacteria in weanling pigs. Arch Anim Nutr. 2003;57:1–10. 10.1080/0003942031000086662. 12801075

[51] Holman DB, Gzyl KE, Mou KT, Allen HK. Weaning age and its effect on the development of the swine gut microbiome and resistome. Msystems. 2021;6:e00682–21. 10.1128/mSystems.00682-21. 34812652 10.1128/mSystems.00682-21 PMC8609972

[52] Gaire TN, Scott HM, Noyes NR, Ericsson AC, Tokach MD, Menegat MB, et al. Age influences the temporal dynamics of microbiome and antimicrobial resistance genes among fecal bacteria in a cohort of production pigs. Anim Microbiome. 2023;5:2. 10.1186/s42523-022-00222-8. 36624546 10.1186/s42523-022-00222-8 PMC9830919

[53] Annamalai T, Saif LJ, Lu Z, Jung K. Age-dependent variation in innate immune responses to porcine epidemic diarrhea virus infection in suckling versus weaned pigs. Vet Immunol Immunopathol. 2015;168:193–202. 10.1016/j.vetimm.2015.09.006. 26433606 10.1016/j.vetimm.2015.09.006 PMC7112776

[54] Jang KB, Purvis JM, Kim SW. Dose–response and functional role of whey permeate as a source of lactose and milk oligosaccharides on intestinal health and growth of nursery pigs. J Anim Sci. 2021;99:skab008. 10.1093/jas/skab008. 33521816 10.1093/jas/skab008 PMC7849970

[55] McLamb BL, Gibson AJ, Overman EL, Stahl C, Moeser AJ. Early weaning stress in pigs impairs innate mucosal immune responses to enterotoxigenic *E. coli* challenge and exacerbates intestinal injury and clinical disease. PLoS One. 2013;8:e59838. 10.1371/journal.pone.0059838. 23637741 10.1371/journal.pone.0059838 PMC3634819

[56] Niekamp SR, Sutherland MA, Dahl GE, Salak-Johnson JL. Immune responses of piglets to weaning stress: impacts of photoperiod. J Anim Sci. 2007;85:93–100. 10.2527/jas.2006-153. 17179544 10.2527/jas.2006-153

[57] McCauley I, Hartmann PE. Changes in the proportion and absolute number of T lymphocytes in piglets from birth until after weaning and in adults. Res Vet Sci. 1984;37:52–7. 10.1016/S0034-5288(18)31927-1. 6332355

