# Supplementary material for: Impacts of different dietary soybean meal levels on jejunal immunity of nursery pigs at different days post-weaning
Source: J Anim Sci Biotechnol. 2025 Oct 24;16:138. doi: 10.1186/s40104-025-01271-0 (PMC12551332; doi:10.1186/s40104-025-01271-0)
Supplement: Supplementary file 1 — Additional file 1: Table S1 Fecal score of nursery pigs fed low or high soybean meal diets. Table S2 Growth performance of nursery pigs fed low or high soybean meal diets. [file 40104_2025_1271_MOESM1_ESM.docx]

**Table S1** Fecal score of nursery pigs fed low or high soybean meal diets^1,2^

| **Item** | **SBM** | | **SEM^3^** | ***P-*value** |
| --- | --- | --- | --- | --- |
|  | **Low** | **High** |  |  |
| Phase 1 (d 0 to 10) | 4.13 | 4.23 | 0.10 | 0.412 |
| Phase 2 (d 10 to 20) | 3.08 | 3.16 | 0.04 | 0.088 |
| Phase 3 (d 20 to 42) | 3.08 | 3.11 | 0.03 | 0.123 |

^1^ Fecal score was recorded during the experiment using a 1 to 5 scale: (1) very firm stool, (2) normal firm stool, (3) moderately loose stool, (4) loose and watery stool, and (5) very watery stool

^2^ Experimental unit was a pig; each treatment had 24 replicates in phases 1 and 2, and 12 replicates in phase 3

^3^ *SEM* Standard error of the mean

**Table S2** Growth performance of nursery pigs fed low or high soybean meal diets^1^

| **Item** | **SBM** | | | **SEM^2^** | ***P-*value** |
| --- | --- | --- | --- | --- | --- |
|  | **Low** | | **High** |  |  |
| Body weight, kg |  | |  |  |  |
| d 0 | 6.2 | | 6.2 | 0.2 | 0.960 |
| d 10 | 7.7 | | 7.1 | 0.2 | 0.004 |
| d 20 | 11.7 | | 11.2 | 0.4 | 0.303 |
| d 42 | 27.6 | | 27.4 | 0.9 | 0.874 |
| Average daily gain, g/d | |  | |  |  |
| Phase 1 (d 0 to 10) | 158 | | 97 | 17 | 0.002 |
| Phase 2 (d 10 to 20) | 402 | | 414 | 31 | 0.707 |
| Phase 3 (d 20 to 42) | 721 | | 739 | 26 | 0.615 |
| Overall | 510 | | 505 | 21 | 0.875 |
| Average daily feed intake, g/d | | | |  |  |
| Phase 1 (d 0 to 10) | 218 | | 172 | 22 | 0.007 |
| Phase 2 (d 10 to 20) | 562 | | 506 | 38 | 0.142 |
| Phase 3 (d 20 to 42) | 1,058 | | 1,036 | 36 | 0.684 |
| Overall | 732 | | 695 | 27 | 0.339 |
| Gain-to-feed ratio | |  | |  |  |
| Phase 1 (d 0 to 10) | 0.79 | | 0.59 | 0.08 | 0.011 |
| Phase 2 (d 10 to 20) | 0.70 | | 0.83 | 0.03 | 0.001 |
| Phase 3 (d 20 to 42) | 0.66 | | 0.70 | 0.03 | 0.142 |
| Overall | 0.68 | | 0.72 | 0.03 | 0.163 |

^1^ Experimental unit was a pig; each treatment had 24 replicates in phases 1 and 2, and 12 replicates in phase 3

^2^ *SEM* Standard error of the mean
